# The Content Validity of an Instrument That Measures Health-Seeking Behavior for Tuberculosis among People Living with HIV in India

**DOI:** 10.3390/tropicalmed9080181

**Published:** 2024-08-16

**Authors:** Ankeeta Menona Jacob, Jeni Jacob, Wim Peersman, Avinash K. Shetty

**Affiliations:** 1 Department of Community Medicine, Nitte (Deemed to be University), KS Hegde Medical Academy, Mangalore 575018, Karnataka, India; dr.ankeetamj_kshema@nitte.edu.in (A.M.J.); jeni.jacob12@gmail.com (J.J.); 2 Faculty of Applied Social Studies, Odisee University of Applied Sciences, 1000 Brussels, Belgium; wim.peersman@odisee.be; 3 Department of Rehabilitation Sciences, Ghent University, 9000 Gent, Belgium; 4 Department of Pediatrics and Office of Global Health, Wake Forest University School of Medicine, Winston-Salem, NC 27157, USA

**Keywords:** content validity, health-seeking behavior, tuberculosis, people living with HIV, India

## Abstract

Determinants of health-seeking behavior among people living with HIV (PLHIV) are important. This study aims to develop and assess the content validity of an instrument that measures health-seeking behavior for tuberculosis among PLHIV in India. An instrument was developed using deductive methods and the Modified Andersen’s Behavioral Model of Health Services (BMHS). We identified three domains using the BMHS. Ten subject experts validated the tool between June 2022 and August 2022. Lawshe’s Content Validity Ratios (CVRs) and Scale Content Validity Indices (CVIs) were computed. The items with CVR and CVI values ≥0.62 were considered for the final tool. The health-seeking behavior among PLHIV, based on the BMHS, identified knowledge regarding TB (domain 1), healthcare accessibility and factors leading to delay in treatment-seeking behavior (domain 2), and client satisfaction regarding diagnostic and treatment-seeking behavior (domain 3). Content Validity Ratios (CVRs) of all the items related to domains 1 and 2 were ≥0.62. The Scale Content Validity Indices (for relevance), i.e., S-CVI (average) and S-CVI (proportional relevance), were ≥0.62 for all domains. The items with CVR and CVI values ≥0.62 in the domains were accepted in the final tool, which contained 69 items.

## 1. Introduction

The Global Tuberculosis Report 2023 estimated that India contributed 27% of global TB cases, affecting more than 2.8 million people. India is one of the eight countries that have the highest number of TB cases globally. The report also estimated that there were 0.34 million TB-related deaths in India and 11,000 deaths among TB/HIV-infected individuals [1]. According to the India Tuberculosis Report 2023 by the World Health Organization (WHO), the TB incidence rate (which represents new cases per 100,000 people per year) was reduced by 16%, and mortality was reduced by 18% compared to 2015 [2].

The poor performance of these indicators for TB was compounded due to the COVID-19 pandemic. The COVID-19 pandemic caused an increase in TB deaths for the first time in the past nine years due to the global decline in TB diagnostic and treatment services [3]. Delays in TB diagnosis among PLHIV can be reduced if the determinants of health-seeking behavior are assessed and addressed promptly among the study population [4]. The Modified Andersen’s Behavioral Model of Health Services (BMHS) identified three groups of factors that influence a person’s decision to seek healthcare. The first one is predisposing factors, which include age, gender, level of education, income, health beliefs, and attitudes about healthcare. The second group is enabling factors, which include transportation, childcare, health insurance, and knowledge of where to receive care. The third group is need-based factors, which include the severity of the perceived problem and the person’s belief that the healthcare system can help them [5]. The Modified Andersen’s BMHS can be used to identify factors that prevent people with TB from seeking care, especially those likely to develop TB, like PLHIV. Since HIV infection contributes significantly to the development of active tuberculosis and TB/HIV co-infection, determining the health-seeking behavior for TB among PLHIV is a decisive factor in reducing morbidity and mortality among TB/HIV co-infected patients [6]. 

A study in Namibia showed that fear, stigma, and discrimination posed barriers to HIV and TB co-infected individuals seeking timely healthcare [6]. A study in Mozambique identified stigma related to diagnosis and treatment, absence of nutritional support, long waiting hours at healthcare facilities, and lack of overall knowledge about TB in the community as barriers to health-seeking among TB/HIV co-infected patients [7]. In India, a study by Samal et al. identified knowledge, perception, and health service provision for TB, delays in health-seeking behavior, and client satisfaction regarding the services in the TB care facilities where there were some barriers to poor health-seeking behavior among PLHIV [8]. However, no validated tool currently exists to determine health-seeking behavior for TB among PLHIV in our settings, which warrants further research. 

The validity of an instrument may be defined as the extent to which it measures what it purports to measure [9]. The content validity measures of the instrument should reflect the population to which this instrument is to be applied. The evaluation of content validity uses a judgmental approach, which includes literature reviews, followed by assessments conducted by expert judges or panels. This process necessitates experts to facilitate the validation process [10]. It is paramount to determine such validity measures, especially when a new instrument has been developed. The measurement of content validity is highly recommended for the initial validation phases as this is an essential step, along with other validity measures [10,11]. 

Content validation undergoes several processes: a thorough literature review, the preparation of a content validation checklist, the selection of a panel of subject experts, depending on the research question, the ascertainment of content validity measures, and the review of domains and items [11]. In the determination of Content Validity, the Content Validity Ratio (CVR) and Content Validity Index (item-level (I-CVI) and scale-level (S-CVI)) are commonly employed. The current study aims to determine the Content Validity Ratio (CVR) and Content Validity Index (item-level (I-CVI) and scale-level (S-CVI)) of the instrument developed for this study to determine the health-seeking behavior for TB among PLHIV.

## 2. Materials and Methods

In this cross-sectional study, we developed and validated an instrument using the Content Validity Indices to determine the health-seeking behavior for TB among PLHIV using the Modified Andersen’s Behavioral Model of Health Services (BMHS) between May 2022 and September 2022.

Step 1: Identification of the domains for the construct and item generation by a thorough deductive literature review

A tool for assessing health-seeking behavior for TB among PLHIV was devised by conducting a comprehensive examination of articles from PubMed Central and Scopus journals using the following MeSh words: TB/HIV, PLHIV, TB, health seeking, health-seeking behavior, barrier, facilitators, delays, HIV stigma, TB stigma, HIV/TB treatment, and HIV/TB co-infection. The categorization of health-seeking behavior into domains was achieved through an extensive literature review of 80 articles using a deductive approach. The literature-generated items were integrated into the Modified Andersen’s Behavioral Model of Health Services (BMHS) domains to conceptualize the construct for health-seeking behavior for TB among PLHIV [5]. The literature review found no existing scales to measure health-seeking behavior for TB among PLHIV in India. This led us to conduct this study to develop a new construct encompassing all the determinants of health-seeking behavior for TB among PLHIV.

Step 2: Categorization of items into dimensions based on the Modified Andersen’s Behavioral Model of Health Services (BMHS)

A systematic framework was created using the modified BMHS, encompassing three domains, as described earlier [5]. These domains included a comprehensive set of 24 items focused on knowledge, symptoms, and the transmissibility of TB (Supplemental Table S1), as well as 31 items dedicated to exploring the health-seeking behavior related to the perception of illness and presumptive tuberculosis symptoms. Additionally, 26 items were designed to evaluate clients’ satisfaction with TB treatment at their preferred health facility. The validation of this instrument occurred within an extensive research endeavor undertaken in a coastal district of Karnataka, India, aimed at investigating the health-seeking behavior patterns of people living with HIV (PLHIV) in relation to TB.

Step 3: Judgment of the scale by expert validation

To ensure accuracy and credibility, a group of ten distinguished experts in TB and HIV were carefully selected and contacted through various means, such as telephone, email, and face-to-face meetings. The panel consisted of five TB specialists (three respiratory medicine experts and two public health specialists who have made significant contributions to the field) and five HIV experts (the medical officer of a district antiretroviral therapy (ART) center, two HIV medicine specialists, and two public health specialists) involved in National HIV Program in India and HIV-related research. Before obtaining written consent for participation, the experts were thoroughly briefed on this study’s purpose, content validation nature, and scope. Written consent and acceptance statements were obtained from each expert before content validation was undertaken. The data were entered into Microsoft Excel (Version 2407), and Content Validity Ratios and Indices were calculated using the Professional version of Microsoft 365 (Version 2407). The experts’ list and their areas of expertise and contributions to the instrument’s content validation are displayed in Table S2 of the Supplementary Materials.

Step 4: Quantification of Content Validity Ratios and Indices

The computation and quantification of Content Validity Ratios and Indices have been described in studies conducted by Zamanzadeh V, Ghahramanian A, Rassouli M, Abbaszadeh A, Alavi-Majd H, and Nikanfar AR [9]. Similar methods were used to derive the Content Validity Ratio and Indices; a cut-off value of ≥0.62 was used based on the number of experts who validated the instrument [9,10].

Step 4a: Quantifying content validity using Lawshe’s score or Content Validity Ratio

**Figure 1:**
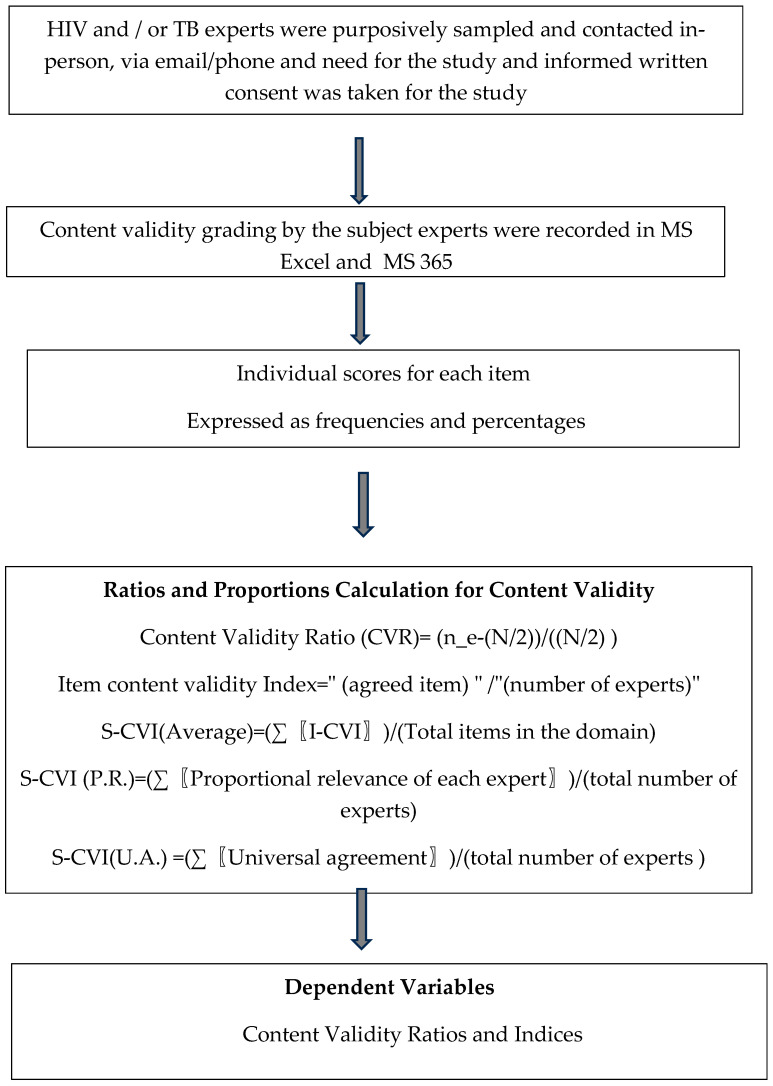
Data acquisition for the content validity of a multidimensional tool to assess health-seeking behavior for tuberculosis among PLHIV.

The Content Validity Ratio (CVR) was calculated for the subject expert responses based on the tool’s usefulness (1—unnecessary, 2—useful but not necessary, and 3—useful/essential). The CVR using Lawshe’s table, containing the CVR cut-offs based on the number of panel experts validating the tool, was ≥0.62 and was considered for inclusion in the final validated tool. The items ≤0.62 were discarded; those with CVRs of <1 but ≥0.62 were modified and included in the final tool, as seen in Figure 1 [11].

I-CVI: Item-wise Content validity Index, S-CVI(Avg): Scale-wise Content Validity Index(average), S-CVI(PR): Scale-wise Content Validity Index (proportional relevance), SCI(UA): Scale-wise Content Validity Index (universal agreement), and PLHIV: People living with HIV. *p*-value <0.05 for values ≥0.62 for 10 subject experts.

Step 4b: Proportional agreement on items and the scale quantification using the Content Validity Indices (CVIs) according to Waltz and Brussel

The subject experts rated the domains in the tools based on relevance using a 4-point Likert scale. The items were graded as 1 = not relevant/clear, 2 = item needs some revision, 3 = clear but needs minor revision, and 4 = very clear by the subject experts. The items for relevance were further used to calculate the CVI for each item and scale-level domains identified. The rest of the clarity, simplicity, and ambiguity points were used to modify and refine the question if the score was ≤3. The content validity indices were calculated for each item using the Item-wise Content Validity Index (I-CVI). The Content Validity Indices were calculated at the scale level using an average CVI (S-CVI(average)), proportional relevance (S-CVI (proportional relevance), and universal agreement among experts (S-CVI (universal agreement)), as seen in Figure 1. The items with Content Validity Ratios and Content Validity Indices of ≤0.62 were eliminated. The items rated ≤ 2 among the experts for clarity, simplicity, and ambiguity were suitably modified and included with CVRs and CVIs ≥0.62 for those specific items [11].

## 3. Results

### 3.1. Design of the Instrument to Measure Health-Seeking Behavior for Tuberculosis among PLHIV

**Figure 2:**
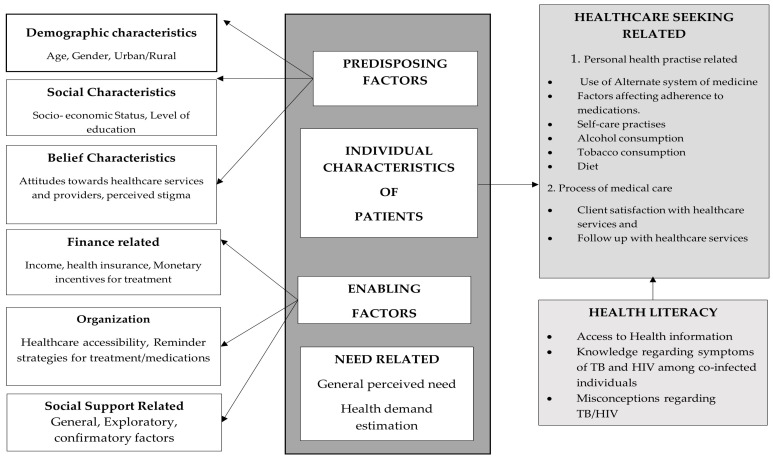
Conceptual framework for health-seeking behavior for tuberculosis among PLHIV using the Modified Andersen’s Behavioral Model of Health Services (BMHS).

A conceptual framework was constructed using the Modified Andersen’s Behavioral Model of Health Services (BMHS), and domains for this study were identified. The explanatory variables were chosen based on the Modified Andersen’s BMHS, which identified individual characteristics of PLHIV as health literacy and healthcare-seeking factors, followed by the enabling and need-based factors. The literature review identified factors overlapping with this model to create the conceptual framework, as shown in Figure 2. 

Since there was no prior research on the health-seeking behavior for tuberculosis, articles on health-seeking behavior for tuberculosis among PLHIV were categorized into three broad domains (Table 1) before conducting quantitative or qualitative data analysis. 

The research questionnaire included two standardized questionnaires: the Medication Adherence Questionnaire (MAQ) and Berger’s HIV Stigma Scale. These questionnaires have already been validated in India. The validation process was conducted for three domains:

- Knowledge of the symptoms and transmission of TB.
- Health care accessibility, barriers, and factors related to delays in seeking treatment for tuberculosis among people living with HIV (PLHIV).
- Client satisfaction regarding diagnostic and treatment-seeking behavior for TB among PLHIV.

### 3.2. Determination of the Content Validity Ratios and Content Validity Indices of the Items in the Scale

Table 2 shows the number of items in each domain with the Content Validity Ratio (CVR). The results showed that all items in the domains were accepted, except for domain 3, which had questions related to client satisfaction. The client satisfaction domain had the highest number of items (12 or 46.2%) that were rated non-essential or not useful (i.e., with CVRs ≤ 0.62) and were not included in the final tool at a *p*-value < 0.05.

Table 3 shows the domain-wise Content Validity Indices (item- and scale-level). The Content Validity Indices (average and proportional relevance) were ≥0.62 for all items included in the final scale. The S-CVI (universal agreement) was also seen among the experts for the items included in domains 1 and 2. However, because the 12 items in domain 3 had poor CVRs, the S-CVI (universal agreement) that affected the scale was rejected and not included in the final scale.

### 3.3. Finalizing the TB Health-Seeking Behavior Scale for PLHIV

Table 4 displays the items’ CVR, I-CVI, and S-CVI scores based on average, proportional relevance, and universal agreement, demonstrating high content validity for the domains. As a result, the final tool comprises 69 items across three domains, with 24 items focused on the knowledge of symptoms and transmission of TB, 31 items related to healthcare accessibility and factors that contribute to delays in seeking treatment for tuberculosis, and 14 items about client satisfaction with diagnostic and treatment services offered by healthcare facilities. Table S1 of the Supplementary Materials contains the items that are included in the final version of the questionnaire.

## 4. Discussion

This research aimed to develop and validate an instrument designed to determine the health-seeking behavior related to TB among PLHIV in India, utilizing the Modified Andersen’s Behavioral Model of Health Services. The findings indicated that this study had a reasonable level of validity, with Content Validity Ratios and Indices of ≥0.62 at *p*-values < 0.05. The final instrument included three categories with 69 items, encompassing 24 items regarding knowledge of TB symptoms and transmission, 31 items on healthcare access and delays in seeking TB treatment, and 14 items on client satisfaction with diagnostic and treatment services within healthcare facilities. Twelve client satisfaction items did not achieve significant CVR levels and were removed from the final tool.

Tolera et al. applied the Modified Andersen’s Behavioral Model of Health Services (BHMS) to investigate antenatal care utilization in a healthcare facility located in Western Ethiopia. This study revealed that programmatic interventions targeting predisposing factors such as religion, number of antenatal care visits, and enabling factors such as household income >$50 and more than two visits by Health Extension Workers (HEWs) were necessary. Additionally, this model identified severe headaches during the antenatal period as a need-based factor [14].

Another study utilized the Modified Andersen’s Behavioral Model of Health Services (BMHS) to examine the behavioral factors associated with hypertension in men who have sex with men. We gained insights into healthcare utilization by identifying vulnerable predisposing factors, need-based factors, and other predisposing factors [15]. Similarly, we employed a TB diagnosis as a surrogate measure of healthcare seeking and utilization of TB services among PLHIV.

Seidu AA conducted a study in Ghana to evaluate the utilization of HIV testing services among sexually active men. Andersen’s model was used to identify specific groups that require programmatic intervention. The study highlighted that unmarried men and men from the Mole-Dagbani ethnic group with lower levels of education were the target groups for intervention [16].

Pampalia et al. conducted a study to assess the knowledge, stigma, and health-seeking behavior of patients co-infected with HIV and TB in Jakarta. They used a questionnaire that consisted of demographic characteristics, HIV-KQ-18, a TB knowledge survey questionnaire, the Berger’s HIV Stigma Scale, and TB-related stigma. Health-seeking behavior was categorized as visiting when the symptoms appeared (≥15 days was a delay or would be poor health-seeking behavior and <15 days was good behavior/less consultation delay) [17]. Onyango et al. conducted a study in South Africa to determine knowledge, attitudes, and health-seeking behavior among patients with TB. They created a questionnaire that drew from prior research as well as advice from TB experts (TB nurses). The questionnaire comprised four components. The demographic profiles (age, gender, education level, marital status, employment, and housing) comprised the main part of Section A. The questionnaire’s portion B focused on TB patients’ knowledge of TB, while section C addressed their attitudes toward the disease. Section D focused on the health-seeking behavior of TB patients. For health-seeking behavior, patients were asked about their perceptions of their early tuberculosis symptoms, usage of alternative treatments, treatment follow-up, and treatment location. To assess TB stigma, participants were asked how TB patients are perceived or supported [18]. The lack of consideration for financial enabling elements in these questionnaires resulted in a superficial understanding of health-seeking behavior. We can consider the enabling factors as a component of health-seeking behavior by applying the Modified Andersen’s Behavioral Model of Health Services.

Burapat et al. assessed health-seeking behavior among HIV-infected patients treated for TB in Thailand by using various factors, as follows: socio-demographic characteristics, high TB stigma, low HIV knowledge, usage of sleeping pills, smoking, alcohol intake, hepatitis B surface antigen and hepatitis C virus infection status, whether they had ever been in prison, men who have sex with men (an at-risk group), IV drug abuse, delay in TB diagnosis, and the location where TB/HIV treatment was sought when they first became sick, which is the most influential factor in choosing the medical provider [19]. We discovered that the aforementioned factors were significant for the evaluation of health-seeking behavior. Using the Modified Andersen’s Behavioral Model of Health Services, we can further classify these factors into individual characteristics, need-based factors, enabling factors, and predisposing factors to ensure that no crucial elements are missed and to provide a more comprehensive overview of health-seeking behavior.

Buregyeya et al. conducted a qualitative study in rural Uganda among caretakers of TB patients, school heads, opinion leaders, and TB patients to determine knowledge, practice, and health-seeking behavior for TB. They determined beliefs about TB causality, high-risk groups for TB, location of TB care, TB knowledge, and TB stigma to be factors affecting health-seeking behavior [20]. These elements account for only a small portion of health-seeking behavior. We may include many additional elements in an organized fashion based on the Modified Andersen’s Behavioral Model of Health Services.

Khan et al. conducted a mixed-method study in Pakistan to assess knowledge, awareness, and health-seeking behavior for TB among TB patients and the general population. They included the location of the first opinion after TB diagnosis, the first person to inform them if they had TB, the source of information regarding TB as a part of the health-seeking behavior [21]. This also seemed to be insufficient for the assessment of health-seeking behavior, and the Modified Andersen’s Behavioral Model of Health Services can give a comprehensive overview for the development of a multidimensional tool to assess the health-seeking behavior.

Annan et al. conducted a study on the health-seeking behavior of TB patients and related factors in the central region of Ghana. They assessed the same factors by including the socio-demographic characteristics of the study population, history of TB among the study participants, service-related barriers influencing TB management, such as time spent (in minutes) from home to the DOTS center, staff attitude at the DOTS center, and other factors, such as the time elapsed before seeking treatment (first delay by the patient), the patient’s reason for seeking treatment at the first location (health facility), the time elapsed before the patient reported to the health facility (second delay), reasons for seeking treatment elsewhere during DOTS and other forms of TB treatment [22]. Although these details provide an outline of health-seeking behavior, some other factors, such as need-related and enabling factors, were also missing.

One of the key strengths of this study is that we carefully selected 10 subject experts in TB and HIV, which helped to ensure the ratings of the items were robust and reliable. However, it is important to note that our study has some limitations. For example, we did not assess face validity measures in the target population before beginning this study. Furthermore, the content validation of the subject experts was based on their opinions and knowledge of HIV prevalence and the socio-demographic characteristics of the study area. Therefore, it is important to exercise caution when using this tool in settings other than the present study area. The items included in each domain were specific to Dakshina Kannada’s unique cultural, economic, and healthcare landscape, limiting generalizability to other regions in India and globally, where differences in occupational distributions, cultural attitudes, healthcare infrastructure, and economic conditions may lead to varying HIV prevalence and profiles. Ensuring accurate estimates of client attitudes in our cultural setting would be crucial while undertaking cultural validation of the tool. 

The unfortunate stigma surrounding PLHIV can lead to obstacles in accessing healthcare for TB diagnosis. Efforts to address the various factors through this validated instrument may contribute to delays in diagnostic and therapeutic services for PLHIV. However, it is important to note that the deductive method employed in these efforts, which relied on a published literature review, may not have captured all the relevant social, cultural, and economic factors that impact health-seeking behavior among these populations and, thus, may not have provided a fully holistic understanding. 

## 5. Conclusions

It is essential to estimate ratios and content validity indicators when developing a tool by applying content validity. A comprehensive process of literature reviews and expert consultations is required for content validity. The tool developed for this study was created using the Modified Andersen’s Behavioral Model of Health Sciences. It was divided into three domains: knowledge of TB symptoms and transmission (24 items), healthcare accessibility (31 items), factors affecting treatment-seeking delays (14 items), and client satisfaction (24 items) concerning diagnostic and treatment facilities. A CVR and CVI of 0.62 or above confirmed each domain’s validity.

## Figures and Tables

**Table 1:** Domains identified using the Modified Andersen’s Behavioral Model of Health Services (BMHS) for the development of a tool for health-seeking behavior for tuberculosis among people living with HIV (PLHIV).

Health Literacy Domain	Healthcare Seeking Domain	Predisposing Factors Domain	Enabling Factors Domain
Knowledge of symptoms and transmission of tuberculosis disease	Medication adherence domain: using Morisky’s medication adherence questionnaire (already validated in India) [12]	Socio-demographic characteristics of PLHIV attending a district antiretroviral (ART) center	Perceived stigma (Berger’s HIV Stigma Scale: already validated in India) [13]
	Client satisfaction with healthcare services for the diagnosis and treatment of tuberculosis		Healthcare accessibility and factors related to delays in treatment-seeking behavior for tuberculosis
24 items	26 items	10 items	31 items

**Table 2:** Domain and item-wise Content Validity Ratio (CVR) of health-seeking behavior for tuberculosis (TB) among people living with HIV (PLHIV).

Domains	Items	Experts (n) who Deemed the Items as Essential	Lawsche’s Score for CVR	Interpretation
Domain 1: Knowledge of symptoms and transmission of TB among PLHIV	24	24	1.0 for all items	All items accepted for the final scale
Domain 2: Healthcare accessibility and factors related to delays in treatment seeking for TB among PLHIV	31	27	1.0 for 27 items	All items accepted for the final scale
			0.9 for 4 items	Accepted with modifications and included in the final scale
Domain 3: Client satisfaction regarding diagnostic and treatment-seeking behavior for TB among PLHIV	26	14	1.0 for 14 items	All items accepted for the final scale
			≤0.62 for 12 items *	Rejected and not included in the final scale

CVR—Content Validity Ratio. * A CVR of ≤0.62 is required for ten experts, with the level of significance at *p* = 0.05.

**Table 3:** Domain and item-wise Content Validity Index (CVI) of health-seeking behavior for tuberculosis (TB) among people living with HIV (PLHIV).

Domains	Number of Items	I-CVI ≥ 0.62	S-CVI (Average)	S-CVI (Proportional Relevance)	S-CVI (Universal Agreement)	Interpretation
Domain 1: Knowledge of symptoms and transmission of TB among PLHIV	24	24	0.991	0.991	0.92	All items accepted for the final scale
Domain 2: Healthcare accessibility and factors related to delays in treatment seeking for TB among PLHIV	31	31	0.987	0.987	0.87	All items accepted for the final scale
Domain 3: Client satisfaction regarding diagnostic and treatment-seeking behavior among PLHIV	26	26	0.917	0.846	0.038	Only 12 items were not accepted as these items affected the Universal Agreement

I-CVI: Item-wise Content Validity Index, S-CVI (Avg): Scale-wise Content Validity Index (average), S-CVI (PR): Scale-wise Content Validity Index (proportional relevance), SCI (UA): Scale-wise Content Validity Index (universal agreement), and PLHIV: People living with HIV. *p*-value < 0.05 for ≥0.62 for 10 subject experts.

**Table 4:** CVRs, I-CVIs, and S-CVIs based on the average, proportional relevance, and universal agreement of the questionnaire.

Domains	Items	CVR	I-CVI	S-CVI (Average)	S-CVI (Proportional Relevance)	S-CVI (Universal Agreement)	Interpretation
Domain 1 Knowledge of symptoms and transmission of tuberculosis	1	1	1	0.991	0.991	0.916	Excellent; can be included in the final tool
	2	1	1				
	3	1	0.9				
	4	1	1				
	5	1	1				
	6	1	1				
	7	1	1				
	8	1	1				
	9	1	1				
	10	1	0.9				
	11	1	1				
	12	1	1				
	13	1	1				
	14	1	1				
	15	1	1				
	16	1	1				
	17	1	1				
	18	1	1				
	19	1	1				
	20	1	1				
	21	1	1				
	22	1	1				
	23	1	1				
	24	1	1				
Domain 2 Healthcare accessibility and factors related to delays in treatment seeking for tuberculosis among PLHIV	1	1	1	0.987	0.987	0.87	Excellent; can be included in the final tool
	2	1	1				
	3	1	1				
	4	1	1				
	5	1	1				
	6	1	1				
	7	1	1				
	8	1	1				
	9	1	1				
	10	1	1				
	11	1	1				
	12	1	1				
	13	1	1				
	14	1	1				
	15	0.8	1				
	16	1	1				
	17	1	1				
	18	0.8	1				
	19	1	0.9				
	20	1	0.9				
	21	1	0.9				
	22	1	1				
	23	1	1				
	24	1	0.9				
	25	1	1				
	26	1	1				
	27	1	1				
	28	1	1				
	29	1	1				
	30	1	1				
	31	1	1				
Domain 3 Client satisfaction regarding diagnostic and treatment-seeking behavior among PLHIV	1	1	1	0.917	0.846	0.038	Excellent; can be included in the final tool
	2	1	0.9				
	3	1	0.9				
	4	1	0.9				
	5	1	0.9				
	6	1	0.9				
	7	1	0.9				
	8	1	0.9				
	9	1	0.9				
	10	1	0.9				
	11	1	0.9				
	12	0.4	0.8				Poor; not included in the final tool
	13	0.2	0.8				
	14	0.2	0.8				
	15	1	0.8				Excellent; can be included in the final tool
	16	0.4	0.8				Poor; not included in the final tool
	17	0.2	0.8				
	18	0.2	0.8				
	19	1	0.8				Excellent; can be included in the final tool
	20	0.6	0.8				Poor; not included in the final tool
	21	0.4	0.8				
	22	0.6	0.8				
	23	1	0.8				Excellent; can be included in the final tool
	24	0	0.8				Poor; not included in the final tool
	25	0	0.8				
	26	0	0.8				

CVR: Content Validity Ratio, I-CVI: Item-wise Content Validity Index, S-CVI (Avg): Scale-wise Content Validity Index (average), S-CVI (PR): Scale-wise Content Validity Index (proportional relevance), SCI (UA): Scale-wise Content Validity Index (universal agreement), and PLHIV: People living with HIV. *p*-value < 0.05 for ≥0.62 for 10 subject experts.

## Data Availability

The original contributions presented in the study are included in the article, further inquiries can be directed to the corresponding author.

## Supplementary Materials

The following supporting information can be downloaded at https://www.mdpi.com/article/10.3390/tropicalmed9080181/s1. Table S1: Domain-wise questions are included for the final tool after validity measurements. Table S2: List of experts and their area of expertise and contribution to the area of expertise involved in the validation of instrument that measures health-seeking behavior for Tuberculosis among People Living with HIV in India.
