# Supplementary material for: The Content Validity of an Instrument That Measures Health-Seeking Behavior for Tuberculosis among People Living with HIV in India
Source: Trop Med Infect Dis. 2024 Aug 16;9(8):181. doi: 10.3390/tropicalmed9080181 (PMC11359263; doi:10.3390/tropicalmed9080181)
Supplement: Supplementary file 1 [file tropicalmed-09-00181-s001.zip › tropicalmed-3064569-supplementary.pdf]

Supplementary Materials

Table S1 Domain-wise questions are included for the final tool after validity measurements.

DOMAIN 1: KNOWLEDGE REGARDING SYMPTOMS AND MODE OF TRANSMISSION OF TUBERCULOSIS

Item Number	Questions	Options	
		Yes	No
1.	Have you ever heard of an illness called Tuberculosis or TB?		
2.	If yes, what is the source of your information? - Television		
3.	Cinema		
4.	Newspapers/magazines		
5.	Posters/hoardings		
6.	Exhibition/mela		
7.	Health workers		
8.	Religious leaders		
9.	School/teachers		
10.	Political leaders		
11.	Community meetings		
12.	Workplace		
13.	Spouse		
14.	Friends/relatives		
15.	According to you, Tuberculosis spreads from one person to another- Through the air when coughing or sneezing by a tuberculosis patient		
16.	Through sharing utensils with a person with Tuberculosis		
17.	Through touching a person with Tuberculosis		
18.	Through sharing food and water with a person with Tuberculosis		
19.	Through sexual contact with a person with Tuberculosis		
20.	Through mosquito bites		
21.	Any other mode(specify)		
22.	Using public toilets or swimming pools		
23.	Can Tuberculosis be cured?		
24.	Does HIV infection increase the risk of contracting Tuberculosis?		

DOMAIN 2 TREATMENT-SEEKING BEHAVIOUR FOR TUBERCULOSIS AMONG PLHIV

Item Number	Questions	Options	
1.	Have you been given Tuberculosis Preventive therapy?	Yes	No
2.	If yes, how long ago have you taken Tuberculosis Preventive Therapy	months	
3.	Have you been ever asked to submit sputum sample for Tuberculosis?	Yes	No
4.	If yes, how long ago was a sputum examination for tuberculosis testing done?	months	
5.	If not, what were the reasons for not testing for Tuberculosis?	Yes	No
	No specific reason/ Did not perceive to have TB		
6.	Inconvenient work hours to test/treat Tuberculosis		

7.	Don't wish to take more medications if diagnosed with TB			
8.	Fear of being diagnosed with Tuberculosis also			
9.	Fear of Side effects of existing medicines for HIV			
10.	No / Dry cough			
11.	Cough due to other conditions (COVID, Tobacco consumption, allergy etc.)			
12.	Unavailability of services for diagnosis of TB in ART centre			
13.	Sample collection for TB takes long			
14.	Diagnosis of TB takes a long time			
15.	Investigations for TB need periodic repetition of tests			
16.	Cost of consultation for TB at the preferred health enter			
17.	Not asked to submit sputum sample for testing			
18.	Initial empirical treatment was given for cough			
19.	Hesitant to undergo complete diagnostic modality as prescribed by the physician			
20.	Cost to travel to take treatment for HIV or TB			
21.	Have you been co-infected with TB?		Yes	No
22.	What was a sputum examination result?	Not Applicable	Positive	Negative
23.	Type of Tuberculosis	Not Applicable	Pulmonary	Extra Pulmonary
24.	When did you develop symptoms of Tuberculosis?		Months	Not Applicable
25.	How many days from developing symptoms were you diagnosed to be co-infected?		Months	Not Applicable
26.	How many days from the development of symptoms were you started on treatment?		Days	Not Applicable

DOMAIN 3: CLIENT SATISFACTION REGARDING THE HEALTHCARE FACILITY SOUGHT FOR TREATMENT OF TUBERCULOSIS SYMPTOMS

Item Number	Questions	Options	
		**Yes**	**No**
1.	Where do you seek medical care when you perceive that you have symptoms of T.B?<br>Government health care facility		
	Private healthcare facility		
	Alternative system of medicine facility		
	NGO's		
	Others		
2.	Which is your preferred choice for medical care when you perceive that you have symptoms of T.B among the above?		
3.	How far is the healthcare facility you seek medical care for your symptoms of T.B? Km		
4.	What is the average time to reach the healthcare facility ____ min/hour?		
5.	What is the average time to meet the doctor in the healthcare facility? ___ min		
6.	Are the doctors in the healthcare facility show you polite behaviour and communication when you perceive that you have symptoms of T.B?	Yes	No

7.	Are the doctors in the healthcare facility give Health information when you perceive that you have symptoms of T.B?	Satisfactory	Unsatisfactory
8.	Are the doctors in the healthcare facility communicate regarding medications for symptoms when you perceive that you have symptoms of T.B?	Clear	Unclear
9.	Are the doctors in the healthcare facility give Follow up on the advice when you perceive that you have symptoms of T.B?	Yes	No
10.	Are the nurses in the healthcare facility show you polite behaviour and communication when you perceive that you have symptoms of T.B?	Yes	No
11.	Are the registration staff in the healthcare facility show you polite behaviour and communication when you perceive that you have symptoms of T.B?	Yes	No
12.	Are the Lab staff in the healthcare facility show you polite behaviour and communication when you perceive that you have symptoms of T.B?	Yes	No
13.	Are the pharmacy staff in the healthcare facility show you polite behaviour and communication when you perceive that you have symptoms of T.B?	Yes	No
14.	Are the Group D staff in the healthcare facility show you polite behaviour and communication when you perceive that you have symptoms of T.B?	Yes	No

Table S2 Table S2: List of experts and their area of expertise and contribution to the area of expertise involved in the validation of instrument that measures health-seeking behavior for Tuberculosis among People Living with HIV in India.

Validator	Designation	Area of expertise	Contribution to the area of expertise
1	Professor, Department of Internal Medicine, KMC Mangalore, Medical Superintendent and medical officer of ICTC at KMC Mangalore.	HIV	He has treated PLHIV patients at the medical college hospital for 20 years.<br>He is the infection control officer for the college hospital's Integrated Counselling and Testing Center.<br>Some of the Publications on HIV include (DOI link):<br>DOI- 10.1016/S1473-3099(21)00449-7<br>DOI-10.2174/18746136-v16-e2112200<br>DOI: 10.1186/s12981-021-00368-6<br>DOI: 10.1186/s12916-020-01876-4
2	Head of Department, Department of Community Medicine, KS Hegde Medical Academy, Mangaluru, Karnataka, India	HIV	Has served as HIV sentinel Surveillance officer for the National AIDS Control Programme for 10 years<br>Some of the Publications on HIV include (DOI link):<br>DOI: 10.1177/2325957415569309
3	Professor, Department of Internal Medicine, Yenepoya Medical College, Mangaluru, Karnataka, India	HIV	He has treated PLHIV patients at the medical college hospital for 20 years.<br>He is the infection control officer for the college hospital's Integrated Counselling and Testing Center.<br>His areas of interest include HIV medicine, clinical ethics and medical education.<br>He is a member of YU Ethics Committee.<br>Some of the Publications on HIV: Perceptions of people living with HIV/AIDS regarding access to health care,<br>April 2014Medicine and Law 33(1):64-73
4	Associate Professor, Department of Community Medicine, Father Mullers Medical College Mangaluru, Karnataka, India	HIV	Completed Dissertation on Pediatric HIV transmission and has publications related to HIV transmission<br>Some of the Publications on HIV include (DOI link):<br>DOI: 10.4103/mjmsr.mjmsr_40_21<br>DOI: 10.1080/09540121.2020.1851018<br>DOI: 10.1016/j.ijid.2016.02.565
5	ART Medical Officer, Wenlock District Hospital, Dakshina Kannada, Mangaluru, Karnataka, India	HIV	Served as the District ART medical Officer for over 15 years, treating more than 10,000 PLHIV in Mangalore city, Dakshina Kannada district.
6	Head of Department, Department of Community Medicine, KAHER's JGMM Medical College and the current State Task Force(STF) chairperson for Karnataka state for the National Tuberculosis Elimination Programme(NTEP)	TB	Active research work in TB Control and contributing as Nodal Officer of the TB Core Committee of the Medical College.<br>Has worked as the Vice-chairperson for the State Task Force (STF) for Karnataka state for the National Tuberculosis Elimination Programme (NTEP erstwhile RNTCP)<br>Some of the Publications on TB include (DOI link):<br>DOI: 10.4103/ijcm.IJCM_114_21<br>DOI: 10.1080/07399332.2020.1837135

7	Head of Department, Department of Respiratory Medicine, Yenepoya Medical College, Mangaluru, Karnataka, India,	TB	Has treated many patients with TB and TB -HIV Co-infection for more than 15 years.<br>Served the college as a nodal officer for NTEP(erstwhile RNTCP) under the State Task Force of Karnataka) for 5 years.<br>Some of the Publications on TB include (DOI link):<br>DOI: 10.4103/idoj.IDOJ_7_18<br>DOI: 10.1016/j.pupt.2018.05.002<br>DOI: 10.1136/bcr-2020-240581
8	Head of Department, Department of Respiratory Medicine, KS Hegde Medical Academy, Mangaluru, Karnataka, India	TB	Has treated many patients with TB and TB -HIV Co-infection for more than 15 years.<br>Served the college as a nodal officer for NTEP(erstwhile RNTCP) under the State Task Force of Karnataka) for 5 years.<br>Some of the Publications on TB include (DOI link):<br>DOI: 10.4103/lungindia.lungindia_475_18<br>DOI: 10.1136/bcr-2020-240581<br>DOI: 10.1016/j.ijtb.2016.01.031<br>DOI: 10.1378/chest.1703668<br>DOI: 10.5582/bst.2012.v6.3.110
9	Head of Department, Department of Community Medicine, Subbaiah Institute of Medical Sciences and Zonal Task Force (STF) chairperson for South India(Zone 1) for the National Tuberculosis Elimination Programme(NTEP) .	TB	Served as the former State Task Force (STF) chairperson for Karnataka state for the National Tuberculosis Elimination Programme (NTEP).
10	Assistant District Tuberculosis Officer, Kasargod, Kerala	TB	Pulmonologist in Kerala Government Health Services, now based in District Tuberculosis Centre as Jr Consultant Respiratory Medicine.<br>Secretary of Mangalore Chest Association for two years and Organized Three state-level CMEs during his tenure.<br>He is presently secretary (founder secretary) of Kasaragod chest Society (R.)
